# Effect of continued folic acid supplementation beyond the first trimester of pregnancy on cognitive performance in the child: a follow-up study from a randomized controlled trial (FASSTT Offspring Trial)

**DOI:** 10.1186/s12916-019-1432-4

**Published:** 2019-10-31

**Authors:** Helene McNulty, Mark Rollins, Tony Cassidy, Aoife Caffrey, Barry Marshall, James Dornan, Marian McLaughlin, Breige A. McNulty, Mary Ward, J. J. Strain, Anne M. Molloy, Diane J. Lees-Murdock, Colum P. Walsh, Kristina Pentieva

**Affiliations:** 1 Nutrition Innovation Centre for Food and Health (NICHE), School of Biomedical Sciences, Ulster University, Coleraine, BT52 1SA Northern Ireland, UK; 2 Northern Health and Social Care Trust, Causeway Hospital, Northern Ireland, UK; 3 Psychology Research Institute, Ulster University, Northern Ireland, UK; 4 Royal-Jubilee Maternity Service, Belfast, Northern Ireland, UK; 5 Institute of Food and Health, University College Dublin, Dublin, Ireland; 6 School of Medicine, Trinity College Dublin, Dublin, Ireland; 7 Genomic Medicine Group, School of Biomedical Sciences, Ulster University, Northern Ireland, UK

**Keywords:** Prenatal folic acid, Pregnancy, Cognitive performance, Child, Randomized controlled trial, Public health, Wechsler Preschool and Primary Scale of Intelligence

## Abstract

**Background:**

Periconceptional folic acid prevents neural tube defects (NTDs), but it is uncertain whether there are benefits for offspring neurodevelopment arising from continued maternal folic acid supplementation beyond the first trimester. We investigated the effect of folic acid supplementation during trimesters 2 and 3 of pregnancy on cognitive performance in the child.

**Methods:**

We followed up the children of mothers who had participated in a randomized controlled trial in 2006/2007 of Folic Acid Supplementation during the Second and Third Trimesters (FASSTT) and received 400 μg/d folic acid or placebo from the 14th gestational week until the end of pregnancy. Cognitive performance of children at 7 years was evaluated using the Wechsler Preschool and Primary Scale of Intelligence (WPPSI-III) and at 3 years using the Bayley’s Scale of Infant and Toddler Development (BSITD-III).

**Results:**

From a total of 119 potential mother-child pairs, 70 children completed the assessment at age 7 years, and 39 at age 3 years. At 7 years, the children of folic acid treated mothers scored significantly higher than the placebo group in word reasoning: mean 13.3 (95% CI 12.4–14.2) versus 11.9 (95% CI 11.0–12.8); *p* = 0.027; at 3 years, they scored significantly higher in cognition: 10.3 (95% CI 9.3–11.3) versus 9.5 (95% CI 8.8–10.2); *p* = 0.040. At both time points, greater proportions of children from folic acid treated mothers compared with placebo had cognitive scores above the median values of 10 (girls and boys) for the BSITD-III, and 24.5 (girls) and 21.5 (boys) for the WPPSI-III tests. When compared with a nationally representative sample of British children at 7 years, WPPSI-III test scores were higher in children from folic acid treated mothers for verbal IQ (*p* < 0.001), performance IQ (*p* = 0.035), general language (*p* = 0.002), and full scale IQ (*p* = 0.001), whereas comparison of the placebo group with British children showed smaller differences in scores for verbal IQ (*p* = 0.034) and full scale IQ (*p =* 0.017) and no differences for performance IQ or general language.

**Conclusions:**

Continued folic acid supplementation in pregnancy beyond the early period recommended to prevent NTD may have beneficial effects on child cognitive development. Further randomized trials in pregnancy with follow-up in childhood are warranted.

**Trial registration:**

ISRCTN ISRCTN19917787. Registered 15 May 2013.

## Background

Folate plays a crucial role in pregnancy and fetal development as it is essential for cell division and tissue growth, by acting as the key co-factor in one-carbon metabolism and therefore required for nucleotide biosynthesis, amino acid metabolism, and numerous methylation reactions [1]. Conclusive scientific evidence has existed for over 25 years that periconceptional folic acid (FA; the synthetic vitamin form) can protect against neural tube defects (NTDs) [2, 3], but apart from the early pregnancy period targeted for preventing NTD, maternal folate may have other roles in offspring health and particularly in relation to neurodevelopment in the child [4–9]. Folate is recognized among key nutrients required for the formation and development of fetal brain [10] owing to its involvement in the proliferation and growth of neuronal cells and the synthesis of neurotransmitters [11]. Experimental evidence from in vivo studies shows that there is active placental transport of folate and elevated total folate concentrations are found in the brain during early fetal development [12, 13], while prenatal folate deficiency was shown in animal models to decrease progenitor cell proliferation, increase apoptosis, and provoke structural changes in the brain [14, 15]. Observational studies in humans have linked self-reported FA supplement use in the first trimester of pregnancy with better cognitive performance [6–9] and specifically with improved vocabulary and verbal skills [8] in children. Of particular note, a large study of almost 40,000 children in the USA found a lower rate of severe language delay in children at 3 years whose mothers reported taking FA in the first trimester of pregnancy [7]. Maternal FA supplement use was also associated with lower risk of child behavioral and emotional problems [16, 17]. One study, however, found no evidence in secondary analysis that maternal FA supplementation up to the eighth gestational week was associated with long-term somatic and mental development in children [18], albeit this was designed to investigate the effect of multivitamin supplementation on NTD risk and therefore focused on very early pregnancy only, possibly explaining the lack of relationship of FA use with child cognition.

Later pregnancy (i.e., 24–42 gestational weeks) is recognized to be a crucial period for brain growth [19], but far fewer human studies have examined maternal folate status at this time in relation to subsequent cognitive performance in childhood. One study over 40 years ago however reported that children born to mothers with a diagnosis of folate-related megaloblastic anemia during the third trimester of pregnancy had abnormal neurodevelopment and lower intellectual abilities compared with infants born to mothers with optimal folate status [4]. Much more recently, a longitudinal study of 256 mother-child pairs linked maternal folate deficiency diagnosed at the start of the second trimester with reduced brain volume in the children at 6–8 years, as measured using magnetic resonance imaging (MRI) [20]. Correspondingly, higher plasma folate concentrations at the 30th gestational week were associated with better cognitive performance in over 500 children in South India sampled at age 9–10 years [5]. However, the evidence is not entirely consistent, with two longitudinal observational studies from Canada and the USA, respectively, reporting no significant associations of maternal folate biomarkers sampled at several time points between the 16th and the 37th gestational weeks with infant neurodevelopment [21] or cognitive performance of children at age 5 years [19].

Although the totality of evidence generally supports an association of maternal folate during pregnancy with neurodevelopment and cognitive performance in the first decade of life, it is not clear if this relationship is causative as the evidence is drawn from observational studies, often relying on FA supplement use in pregnancy as reported by the mother; information typically collected retrospectively (at time of assessing the child) and thus with a high risk of recall bias. Therefore, it is uncertain whether there are any benefits for the offspring brain arising from continued maternal folic supplementation beyond the first trimester. Accordingly, we conducted a follow-up study of children whose mothers had participated in a randomized controlled trial (RCT) of FA during trimesters 2 and 3 of pregnancy to investigate the effect of maternal FA supplementation on the subsequent cognitive performance in the child.

## Methods

### Study population

**Fig. 1:**
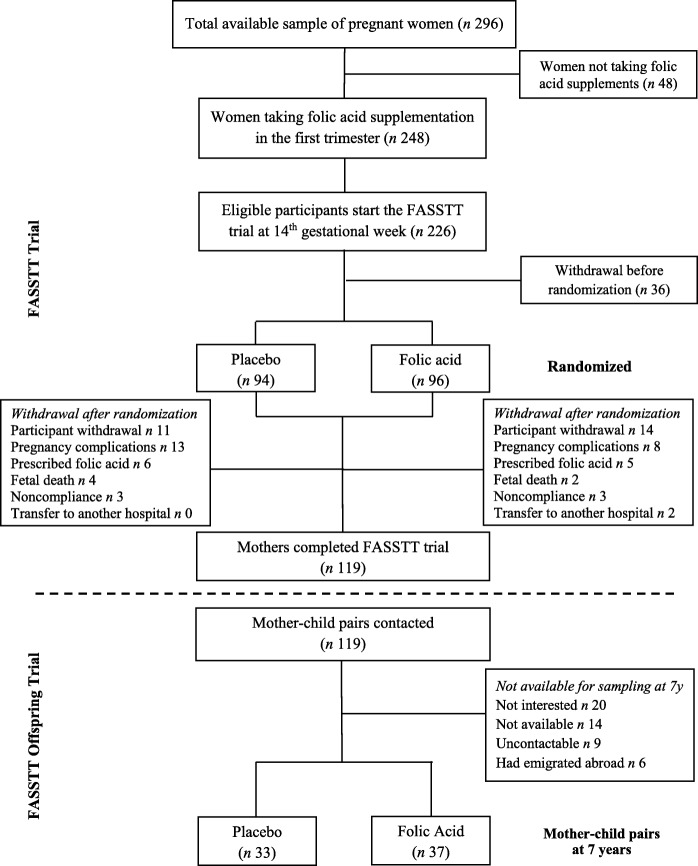
Flowchart showing the study population

This was a follow-up investigation of children whose mothers took part in the Folic Acid Supplementation during the Second and Third Trimesters (FASSTT) trial in pregnancy. The original FASSTT trial conducted in 2006/2007 has been described in detail elsewhere [22]. Briefly for the purpose of this report, healthy pregnant women, aged 18–35 years with a singleton pregnancy and who had taken the recommended dose of 400 μg/d of FA in the first trimester, were recruited from antenatal clinics at the 14th week of gestation (Fig. 1). At the start of the second trimester, eligible participants were randomly assigned to take 400 μg/d FA or placebo for 26 weeks, of which 59 women in the treatment group and 60 in the placebo group completed the trial. FA supplements were distributed (in 7-day pillboxes) to participants every 4 weeks. Based on the recording of unused tablets, an overall participant compliance rate of 93% was estimated.

The FASSTT Offspring Trial was approved by the Office for Research Ethics Committees Northern Ireland and was registered (ISRCTN19917787). Participants from the original FASSTT trial were sent an invitation letter, and in a follow-up telephone call, those who verbally consented to take part were given an appointment to attend the *Nutrition Innovation Centre for Food and Health* at Ulster University. In compliance with ethical requirements, signed written consent from the mother and assent from the child were obtained at the time of the appointment. All efforts were made to recruit the maximum number of participants from the original FASSTT trial. If current contact details were not readily available from our records, the new participant details were traced through health records. Those who failed to attend their appointment were offered an alternative date (up to a maximum of five appointments).

### Measurements of the FASSTT Offspring Trial

#### Cognitive tests

Cognitive performance of the children at age 7 years was completed by a trained researcher using the Wechsler Preschool and Primary Scale of Intelligence test 3rd UK edition (WPPSI-III). This validated clinical instrument for assessing children (up to age 7 years and 3 months) provides composite scores representing intellectual functioning in specified cognitive domains (i.e., verbal IQ, performance IQ, and processing speed) and an overall score for full scale IQ. Each assessment lasted 40–50 min and was completed by the same researcher in one session. To provide an appropriate environment for assessing the child, the room was well-lit, ventilated, and free from distractions, with the researcher seated directly opposite the child and the mother outside of the child’s view. Both researcher and participants were blinded to treatment allocations in the original FASSTT trial.

Prior to conducting the main assessment of the children at 7 years, a pilot investigation of participants at 3 years was conducted using the Bayley’s Scales of Infant and Toddler Development 3rd Edition (BSITD-III), the most frequently used developmental test for infants and young children of 1–42 months, which includes an assessment of cognitive performance, along with other developmental domains such as receptive and expressive communication, and fine and gross motor skills.

#### Anthropometric measurements of the child

Height, weight, waist and head circumference, and body fatness were measured using standardized equipment on the day of the appointment. Height (cm) was measured using a portable, standalone stadiometer, with shoes removed. Weight (kg) was measured to the nearest 0.1 kg using electronic weighing scales, with any heavy clothing removed. Waist and head circumference (cm) were measured using a non-elastic measuring tape. Body mass index (BMI) was calculated as weight in kilograms/height in meters squared. *Z*-scores for BMI were calculated based on the Centre for Disease Control Growth Charts and using the LMS method, which involves the equation *Z* = ((BMI/*M*)^L − 1)/(*L* × *S*) [23]. The parameters of the formula include *M* which is the median BMI by age, *S* which corresponds to the coefficient of variation of BMI, and *L* which allows for age-dependent skewness in the distribution of body mass index [24]. For children aged 7 years, body fat measures were also obtained using the Tanita-305 body fat analyzer (Tanita Corp, Tokyo, Japan).

### Laboratory assessment

Non-fasting blood samples, collected from the mother at 14th and 36th gestational weeks and umbilical cord blood at delivery, were analyzed for serum and red blood cell (RBC) folate by microbiological assay [25]. Methylenetetrahydrofolate reductase (*MTHFR*) C677T genotype was identified using polymerase chain reaction amplification, followed by *Hin*fI digestion [26].

### Statistical analyses

Estimation of the sample size for the current study was based on our pilot investigation in 39 children sampled at 3 years, where assessment scores for the cognitive domain in the BSITD-III test from children of mothers supplemented with folic acid or placebo were used. A sample size of 43 children in each group was estimated for the assessment at 7 years to detect a significant difference in cognitive performance between children of the mothers randomized to each treatment with a power of 80% at α = 0.05 [27].

Statistical analysis was performed using the Statistical Package for the Social Sciences software (version 21.0; SPSS UK Ltd. Chertsey, UK). For normalization purposes, variables were log transformed before analysis, as appropriate. Differences between placebo and treatment groups were analyzed using independent *t* tests for linear variables and chi-square tests for categorical variables. Raw cognitive scores were standardized for the child’s age at time of testing, and appropriate age-specific reference intervals were applied in adherence with test protocols. Analysis of covariance (ANCOVA) was used to test for differences in cognitive variables between treatment groups, with adjustment for relevant confounding factors, including maternal age and education attainment [28], child’s sex [29, 30], birth weight [31], and breastfeeding [28]. Multiple linear regression analysis was used to examine the predictors of cognitive performance in children at 7 years. Further analysis compared mean WPSSI-III composite tests scores for FASSTT Offspring Trial participants with test scores from a nationally representative sample of British children using a one-sample *t* test. The latter cohort, collected for the UK WPPSI-III Project, was specifically sampled to validate this test for use in the UK. During 2002–2003, children were sampled to represent the UK population according to the 2001 Census, in terms of geographic regions, sex, age, ethnicity, and parental educational level [32]. Of a total of 805 children of age 2 years 6 months to 7 years 3 months sampled for this validation project, the mean WPSSI-III scores for 41 children at age 7 years were used in the current analysis.

## Results

Of 119 participants in the original FASSTT trial, 70 mother-child pairs completed the FASSTT Offspring Trial at 7 years, representing a 59% response rate. Of the 70 children sampled, 50% (*n* = 34) was previously also sampled for the pilot study at 3 years, along with 5 other children who were not available for the 7-year follow-up (providing a total of 39 children for assessment at 3 years). A comparison of responders and non-responders to follow-up at 7 years showed no significant differences in any general characteristic, including maternal age (*p* = 0.207), weeks of gestation at labor (*p* = 0.587), parity (*p* = 0.198), birth weight (*p* = 0.642), Apgar score at 5th minute (*p* = 0.760), or % breast fed from birth (*p* = 0.415). Likewise, there were no significant differences in general characteristics between treatment groups among non-responders (Additional file 1: Table S1). No adverse events were reported at any time during the FASSTT trial or at either of the follow-up phases of the study.

### Study cohort characteristics and folate biomarkers in mothers and newborns

There were no significant differences between the placebo and treatment groups in general maternal or child characteristics (Table 1 and Additional file 1: Table S2). Pre-intervention (i.e., at the 14th gestational week), neither serum nor RBC folate, showed significant differences between the treatment groups, but both biomarkers were significantly higher in the FA group compared with placebo following intervention (Table 2). Analysis of the cord blood at delivery also showed significantly higher folate concentrations in the FA treatment group. The frequency of the *MTHFR* 677TT genotype (variant genotype for a common folate polymorphism) was not significantly different between the treatment groups among mothers or children.

**Table 1:** General characteristics of FASSTT Offspring Trial participants at age 3 and 7 years

	Participants at 3 years (*n* = 39)		Participants at 7 years (*n* = 70)	
	Placebo (*n* = 16)	Folic acid (*n* = 23)	Placebo (*n* = 33)	Folic acid (*n* = 37)
Maternal characteristics				
Age, years^a^	27.1 (25.1, 29.0)	28.8 (27.2, 30.4)	28.0 (26.4, 29.6)	29.4 (28.2, 30.7)
BMI, kg/m^2^	25.0 (22.9, 27.1)	25.7 (22.8, 28.6)	24.3 (23.1, 25.6)	25.3 (23.5, 27.2)
Smoking in pregnancy, %	38	22	12	19
Alcohol use, %	6	0	3	3
Duration of folic acid use at time of enrolment, weeks	13.5 (8.4, 18.6)	12.0 (9.0, 15.0)	13.6 (10.7, 16.4)	12.8 (10.5, 15.1)
Iron supplement use, %	25	22	18	27
Married, %	73	91	88	95
Education attainment, years^b^	19.2 (17.8, 20.6)	19.7 (18.5, 20.9)	19.6 (18.5, 20.6)	19.5 (18.5, 20.4)
Homeowner, %	60	76	79	76
Parity (*n*)	2.9 (2.5, 3.3)	3.2 (2.7, 3.6)	2.6 (2.3, 2.9)	2.8 (2.5, 3.1)
Week of gestation at labor	40.0 (39.3, 40.7)	40.0 (39.3, 40.6)	40.2 (40.0, 40.7)	39.9 (3.94, 40.3)
Child characteristics at birth				
Sex, female %	75	65	48	59
Birth weight, g	3392 (3144, 3640)	3317 (3029, 3605)	3519 (3323, 3716)	3374 (3188, 3559)
Birth length, cm	51.1 (49.9, 52.4)	50.6 (49.6, 51.7)	51.4 (50.4, 52.4)	50.8 (50.1, 51.5)
Head circumference, cm	34.4 (33.6, 35.2)	34.4 (33.6, 35.2)	34.6 (34.1, 35.2)	34.6 (34.1, 35.1)
Apgar at 1st minute	8.3 (7.6, 8.9)	8.5 (8.2, 8.8)	8.5 (8.1, 8.9)	8.7 (8.5, 8.8)
Apgar at 5th minute	9.0 (9.0, 9.0)	9.0 (8.9, 9.1)	9.0 (8.8, 9.1)	9.0 (8.9, 9.1)
Breastfed from birth, %	56	44	39	46
Child characteristics at follow-up				
Age at assessment, years	2.8 (2.5, 3.0)	2.7 (2.6, 2.9)	6.8 (6.7, 6.9)	6.7 (6.7, 6.8)
BMI *Z*-score^c^	0.55 (− 0.39, 1.48)	0.19 (− 0.19, 0.57)	0.24 (− 0.11, 0.58)	− 0.08 (− 0.40, 0.23)

Continuous measures presented as mean (95% CI), unless otherwise indicated. Categorical measures compared using Pearson’s chi-square, as appropriate

^a^Age of mother at enrolment to the FASSTT trial

^b^Age of leaving of formal education

^c^Body mass index (BMI) was calculated as weight in kilograms/height in meters squared. BMI *Z*-score was calculated based on the Centre for Disease Control Growth Charts and using the LMS method, using the equation *Z* = (BMI/*M*)^*L* − 1)/(*L* × *S*) [23]. The parameters of the formula include the following: *M* which is the median BMI by age, *S* which corresponds to the coefficient of variation of BMI, and *L* which allows for age-dependent skewness in the distribution of body mass index [24]

**Table 2:** Maternal and cord blood folate biomarkers of FASSTT trial participants

	Placebo (*n* = 33)	Folic acid (*n* = 37)	*p* value
14th GW (pre-intervention)			
Serum folate, nmol/L	48.7 (40.7, 56.7)	45.6 (39.1, 52.1)	0.544
RBC folate, nmol/L	1109 (846, 1371)	1223 (1025, 1421)	0.312
36th GW (post-intervention)^a^			
Serum folate, nmol/L	26.0 (18.9, 33.2)	51.1 (43.6, 58.6)	< 0.001
RBC folate, nmol/L	978 (823, 1133)	1834 (1609, 2060)	< 0.001
*MTHFR* 677TT genotype, %	9	16	0.595
Cord blood^b^			
Serum folate, nmol/L	71.7 (60.5, 83.0)	99.1 (86.6, 111.6)	0.002
RBC folate, nmol/L	1535 (1260, 1810)	2177 (1779, 2574)	0.009
*MTHFR* 677TT genotype, %	14	9	0.914

Continuous measures presented as mean (95% CI) unless otherwise indicated. Continuous measures compared using independent sample *t* test. Count measures compared using Pearson’s chi-square

^a^Post-intervention, following supplementation with folic acid (400 μg/d) for 22 weeks in pregnancy

^b^Cord blood collected upon delivery

### Effect of maternal folic acid during pregnancy on offspring cognition

WPPSI-III composite test scores from children at age 7 years are presented in Table 3. Following adjustment for child’s sex, birth weight, breastfeeding, maternal age, and maternal education attainment, analysis showed that children born to mothers who had received FA in pregnancy scored significantly higher in word reasoning compared to children from the placebo group (mean (95% CI) 13.3 (12.4–14.2) vs 11.9 (11.0–12.8), *p* = 0.027). No other statistically significant differences in WPPSI-III scores were observed between the two groups (results of all subtests are provided in Additional file 1: Table S3).

**Table 3:** WPPSI-III test scores of FASSTT Offspring Trial participants at 7 years

Composite and subtest scores	Placebo (*n* = 33)	Folic acid (*n* = 37)	Difference	*p* value (unadjusted)^a^	*p* value (adjusted)^b^
Verbal IQ	103.4 (99.4, 107.4)	107.7 (103.7, 111.8)	4.3 (− 1.2, 9.9)	0.126	0.120
Information	10.9 (9.9, 11.8)	11.1 (10.3, 12.0)	0.3 (− 1.0, 1.5)	0.648	0.630
Vocabulary	9.6 (8.9, 10.4)	10.3 (9.5, 11.1)	0.7 (− 0.4, 1.8)	0.221	0.262
Word reasoning	11.9 (11.0, 12.8)	13.3 (12.4, 14.2)	1.4 (0.2, 2.6)	0.023	0.027
Performance IQ	100.6 (96.5, 104.6)	104.1 (99.1, 109.1)	3.5 (− 2.9, 9.8)	0.274	0.429
Processing speed	103.9 (98.1, 109.7)	102.5 (97.4, 107.7)	1.4 (− 6.2, 8.9)	0.718	0.712
General language	105.8 (101.1, 110.5)	108.9 (104.5, 113.2)	3.1 (− 3.2, 9.4)	0.334	0.514
Full scale IQ	103.5 (99.3, 107.6)	106.4 (101.7, 111.1)	3.0 (− 3.3, 9.2)	0.348	0.441

Data presented as mean (95% CI)

Test scores assessed by Wechsler Preschool and Primary Scale of Intelligence test, 3rd UK edition (WPPSI-III)

Data analyzed by ^a^independent *t* test and ^b^ANCOVA, with adjustment for maternal age and education attainment [28], child’s sex [29, 30], birth weight [31], and breastfeeding [28]. Results considered significant when *p* < 0.05

When compared with a nationally representative sample of British children at age 7 years, WPPSI-III scores were found to be higher in children from FA treated mothers for verbal IQ (107.7 vs 99.1, *p* < 0.001), performance IQ (104.1 vs 98.7, *p* = 0.035), general language (108.9 vs 101.8, *p* = 0.002), and full scale IQ (106.4 vs 98.3, *p* = 0.001) (Table 4). Comparison of the placebo group with British children however showed smaller differences in scores for verbal IQ (103.4 vs 99.1, *p* = 0.034) and full scale IQ (103.5 vs 98.3, *p* = 0.017) and no differences in performance IQ or general language scores. In neither the FA nor the placebo group were scores for processing speed found to be different from the UK mean scores for children of this age.

**Table 4:** Comparison between WPPSI-III test scores of FASSTT Offspring Trial participants with a representative sample of British children

Composite scores	UK mean (*n* = 41)	Placebo (*n*=33)	*p* value	Folic acid (*n* = 37)	*p* value
Verbal IQ	99.07	103.4	0.034	107.7	< 0.001
Performance IQ	98.74	100.6	0.361	104.1	0.035
Processing speed	101.32	103.9	0.373	102.5	0.636
General language	101.85	105.8	0.098	108.9	0.002
Full scale IQ	98.31	103.5	0.017	106.4	0.001

Data presented as mean. The *p* values refer to data analyzed by a one-sample *t* test for comparison of placebo and treatment groups with WPPSI-III test scores from a representative sample of British children [32]. Results considered significant when *p* < 0.05

Maternal biomarker status of folate at the 36th gestational week was found to be a significant predictor of subsequent verbal IQ (but not other WPPSI-III scores) in children at 7 years: *β* = 0.268 (95% CI 0.000–0.001), *p =* 0.027, for serum folate, after adjustment for relevant covariates, namely maternal age [28], maternal education attainment [28], child’s sex [29, 30], and breastfeeding [28] using multiple linear regression analysis (Additional file 1: Table S4). In this analysis, apart from maternal folate status at the 36th gestational week, breastfeeding was the only factor significantly related to child cognition as determined by WPPSI-III test scores for verbal IQ (*β* = 0.300 (95% CI 0.005–0.053), *p =* 0.017), general language (*β* = 0.369 (95% CI 3.387–16.142), *p =* 0.003), and full scale IQ (*β* = 0.314 (95% CI 1.622–14.837), *p* *=* 0.016).

In the sample of children assessed also at age 3 years, those whose mothers received FA treatment during pregnancy scored significantly higher in the cognitive domain of the BSITD-III test compared to children from placebo mothers (Table 5). No significant differences between the treatment groups in any other developmental domain of the BSITD test were observed.

**Table 5:** Developmental scores of FASSTT Offspring Trial participants at 3 years

Developmental domains	Placebo (*n* = 16)	Folic acid (*n* = 23)	Difference	*p* value (unadjusted)^a^	*p* value (adjusted)^b^
Cognitive^a^	9.5 (8.8, 10.2)	10.3 (9.3, 11.3)	0.8 (− 0.5, 2.1)	0.223	0.040
Receptive communication	10.3 (9.0, 11.5)	10.5 (9.4, 11.6)	0.2 (− 1.4, 1.8)	0.775	0.395
Expressive communication	11.3 (10.0, 12.5)	10.3 (9.1, 11.5)	1.0 (− 0.7, 2.7)	0.257	0.634
Fine motor	9.8 (8.9, 10.7)	10.4 (9.1, 11.6)	0.5 (− 1.1, 2.2)	0.515	0.302
Gross motor	8.6 (7.5, 9.6)	8.6 (7.7, 9.4)	0.0 (− 1.3, 1.3)	0.997	0.828

Data presented as mean (95% CI)

Developmental scores assessed by the BSITD-III

Data analyzed by ^a^independent *t* test and ^b^ANCOVA, adjusting for maternal age and education attainment [28], and child’s birth weight [31]. Results considered significant when *p* < 0.05

**Fig. 2:**
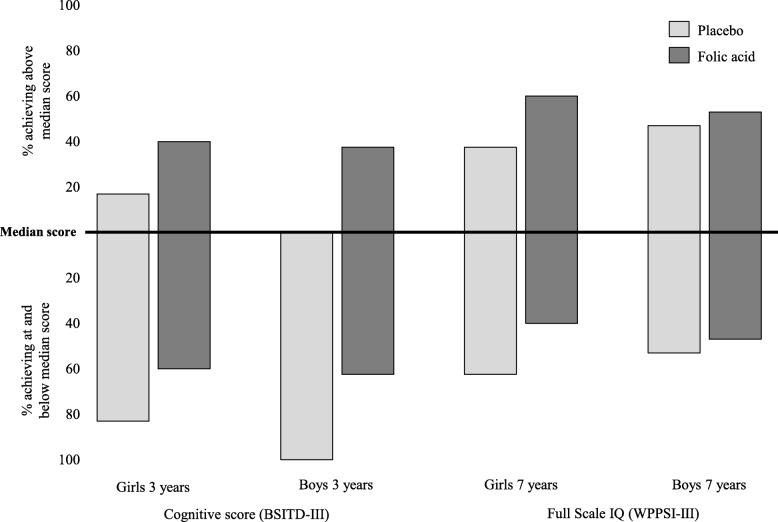
Percentage of FASSTT Offspring participants at 3 and 7 years achieving above average cognitive performance. In children at 3 years, cognitive performance was assessed by the BSITD-III test and at 7 years by the WPPSI-III test. Values are shown as % of children at 3 or 7 years, by treatment group of the mother, who achieved above the median cognitive score for same-sex children at that age versus those who scored at and below the median scores of 10 (for girls and boys) in the BSITD-III test; 24.5 (for girls) and 21.5 (for boys) in the WPPSI-III test. Total numbers at each age and sex group are as follows: girls at 3 years, *n* = 27; boys at 3 years, *n* = 12; girls at 7 years, *n* = 38; and boys at 7 years, *n* = 32

In both assessments (performed at 3 and 7 years), greater proportions of girls and boys from folic acid treated mothers compared with placebo had cognitive scores above the median value of 10 (girls and boys) for the BSITD-III, and 24.5 (girls) and 21.5 (boys) for WPPSI-III (Fig. 2).

## Discussion

As a follow-up study of children whose mothers had participated in an RCT during trimesters 2 and 3 of pregnancy, the FASSTT Offspring study provides the first randomized trial evidence that continuing FA supplementation throughout pregnancy, well beyond the early period known to be protective against NTDs, can influence cognitive development of the child up to 7 years of age. Using validated and internationally recognized tools to measure cognitive performance in children, we show that the children of FA treated mothers during pregnancy had a higher score in the cognitive domain of developmental assessment at 3 years and in word reasoning testing at 7 years. When compared with a nationally representative sample of British children at age 7 years, children from FA treated mothers scored higher in verbal IQ, general language, and full scale IQ.

Apart from its well-established role in preventing NTD in very early pregnancy, folate is known to have other essential roles throughout pregnancy, with impacts in early life and beyond. Folate after the first trimester of pregnancy is likely to be essential for the developing brain as areas such as the hippocampus, striatum, and auditory and visual cortices are undergoing rapid growth to become functionally active at this time [33]. There is evidence that children of mothers diagnosed in late pregnancy with megaloblastic anemia (owing to severe folate deficiency) had abnormal neurodevelopment and lower intellectual abilities [4]. Likewise, previous studies reported reduced psychomotor and cognitive ability, or hyperactivity and peer problems, in children of mothers with low biomarker folate status at preconception or at the 14th gestational week of pregnancy [34, 35]. Other studies have linked higher maternal folate status or self-reported FA use in early pregnancy with improved cognitive performance [6–9] and behavior [16, 17] or reduced risk of severe language delay [7] in children. Despite differences in study design, timing of maternal sampling during pregnancy, and the tests used to assess cognition, the current findings are in broad agreement with the aforementioned studies. The majority of previous studies have however relied on records of pregnancy usage of FA supplements as reported by mothers, and have predominantly focused on the first trimester of pregnancy where official recommendations for FA supplementation to prevent NTD are in place worldwide. In contrast to most previous studies, our study focused on the effects of FA after the period recommended for NTD prevention (but in women who had taken FA in the first trimester, as per the original FASSTT study design), and the findings now provide convincing evidence that better folate status throughout pregnancy may lead to improved cognitive health outcomes in childhood.

The wider public health relevance of our findings is suggested by the supporting data from comparison of the cognitive performance of the FASSTT Offspring Trial participants with that of a nationally representative sample of British children of the same age [32]. When compared with British children at age 7 years, WPPSI-III scores for verbal IQ, general language, and full scale IQ were each higher in children from FA treated mothers. Furthermore, the consistency of our results in relation to cognitive function as measured at 3 years and at 7 years, despite the use of different assessment tools at these time points (and smaller sample at 3 years), strengthens our findings. At both time points, greater proportions of children from folic acid treated mothers compared with placebo achieved cognitive scores above the median value for same-sex children at that age. The cognitive domain of the BSITD-III and the word reasoning test of the WPPSI-III tool measure similar aspects of the brain including verbal comprehension, concept formation, and sensorimotor skills. Our results therefore indicate that the effect of maternal folate may be specific to the verbal domain and not across the broad range of cognition assessed at 7 years, or across developmental domains other than cognitive performance assessed at 3 years. Our findings on cognition are important, not only because achievement of full cognitive potential of every child is considered paramount for future academic attainment, but also because evidence suggests that higher intelligence in childhood is a prerequisite for better cognitive reserves in adulthood that could in turn help to delay cognitive decline in later life [36–38].

The biological mechanisms explaining our findings are not clear. They are likely however to relate to the role of folate within one-carbon metabolism and specifically folate-mediated alterations in methylation which would result in differential expression of proteins related to production of neurotransmitters, myelination, or synaptic formation in the central nervous system [39, 40]. The developing brain is particularly vulnerable to these folate-dependent reactions, and thus, low folate during pregnancy could impair optimal brain development. Furthermore, epigenetic modifications in utero can affect offspring health in later life, with emerging evidence showing that maternal folate can exert epigenetic effects in pregnancy via DNA methylation which could in turn underlie fetal programming and fetal brain development [40–42]. We previously reported folate-mediated epigenetic changes in genes related to brain development and function in the current FASSTT Offspring cohort when they were newborns [43, 44], and this potentially offers a plausible biological basis to link maternal folate during pregnancy with the cognitive effects in childhood found in this study. In this regard, however, the question of optimal FA dose for beneficial effects is somewhat unclear. The presence of plasma unmetabolized FA is reported to arise from higher dose FA supplements in pregnant and non-pregnant women [45]; however, it remains to be established whether there are any associated adverse metabolic or clinical impacts. One recent prospective cohort study (*n =* 2213) showed that 1-year-old children born to mothers reporting to consume FA doses of 5000 μg/d or greater had lower psychomotor development compared to those of mothers who consumed doses of 400–1000 μg/d [45], whereas another study observed beneficial effects on neurodevelopment in 18-month-old children of 5000 μg/d FA taken in early pregnancy compared with no supplementation [8]. Therefore, the effects of exposure to high-dose FA in pregnancy on outcomes in the offspring are unclear and require further investigation. In the meantime, given the uncertainty regarding long-term effects of exposure to high-dose FA, it seems prudent to recommend doses no higher than those demonstrated here in later pregnancy, and for NTD prevention in early pregnancy, as being beneficial with no known harmful effect [46, 47].

A number of factors contribute to the strength of this study. The study design involving the follow-up of children from participants in an RCT in pregnancy [22] enabled us to demonstrate a causative link between maternal FA supplementation and subsequent cognition in the child. Maternal and newborn responses to FA intervention were measured by RBC folate, which is unaffected by recent intake and widely considered to be the best biomarker of long-term folate status [48]. The use of internationally recognized tools to measure cognitive performance in children is also a strength and enables the results from this maternal intervention with folic acid to be placed in a wider public health context for consideration along with findings from other antenatal or child interventions in relation to cognition in children [49, 50]. The main study outcomes in children sampled at 7 years are supported by the pilot data from the same children sampled at 3 years, also showing a beneficial effect of maternal FA on child cognition. Furthermore, the two sampling time points provided the opportunity to track cognitive development into childhood and the broad agreement in results at 3 and 7 years contributes some degree of internal validation to our findings. In addition, in our analysis, we controlled for common confounders including maternal age and education attainment, child’s sex, birth weight, and breastfeeding, previously reported to be strongly associated with child neurodevelopment [28–31]. This study was however not without limitations, the most significant of which was the relatively small sample size. Of 119 participants in the original FASSTT trial, 70 (59%) of the mother-child pairs completed the FASSTT Offspring Trial at 7 years; of these, 34 children were also sampled at 3 years and provided pilot data in relation to child cognition. Although we made every effort to maximize the participation rate from the original FASSTT trial, our final sample may have lacked sufficient statistical power to detect small effects in some test components of the WPPSI-III test. In addition, the sample may not be representative of children generally, in terms of ethnicity and socioeconomic status, and therefore, the results require confirmation in other populations. Future work in this area would be much enhanced by combining cognitive tests as used in the current study with non-invasive brain imaging or novel brain mapping techniques, as previously applied to study the effects of nutritional interventions in pregnancy on brain health outcomes in the child [51, 52].

## Conclusions

In summary, the current findings provide the first randomized trial evidence that continued FA supplementation of mothers through the second and third trimesters of pregnancy can influence the cognitive performance of their children up to 7 years of age. The results show that there are benefits for the child of continuing maternal use of FA throughout pregnancy, whereas current recommendations in most countries worldwide advise mothers to take FA supplements from before conceiving until the end of the 12th gestational week only. If confirmed by further randomized trials in pregnancy with follow-up in childhood, these findings could have important impacts in informing future policy and practice in relation to FA recommendations in pregnancy.

## Supplementary information

**Additional file 1: Table S1a.** Characteristics of responders and non-responders to participation in the FASSTT Offspring trial at 7 years. **Table S1b.** Characteristics of non-responders to participation in the FASSTT Offspring trial at 7 years by treatment group. **Table S2.** Anthropometric measurements of FASSTT Offspring trial participants at age 3 and 7 years. **Table S3.** WPPSI -III test scores of FASSTT Offspring trial participants at 7 years. **Table S4a.** Maternal serum folate status at 36th GW and WPPSI-III test scores of FASSTT Offspring trial participants at 7 years. **Table S4b.** Maternal RBC folate status at 36th GW and WPPSI-III test scores of FASSTT Offspring trial participants at 7 years.

## Data Availability

Data from this study are held in full compliance with Ulster University’s Research Governance and Ethics Policy for Human Research (2018) (https://internal.ulster.ac.uk/research/office/rofficeeg.php), which in turn is fully aligned with the UK’s Data Protection Act 2018. The participants of FASSTT and FASSTT Offspring Trials did not provide consent for sharing their data publicly. Data are available from the Research Governance of Ulster University (UK) for researchers who meet the criteria for access to confidential data. Please address requests to Mr. Nick Curry, Head of Research Governance at Ulster University at n.curry@ulster.ac.uk

## Abbreviations

ANCOVA: Analysis of covariance
BMI: Body mass index
BSITD-III: Bayley’s Scales of Infant and Toddler Development 3rd Edition
FA: Folic acid
FASSTT: Folic Acid Supplementation during the Second and Third Trimesters
GW: Gestational weeks
IQ: Intelligence quotient
MTHFR: Methylenetetrahydrofolate reductase
NTDs: Neural tube defects
RBC: Red blood cell
RCT: Randomized controlled trial
SPSS: Statistical Package for the Social Sciences software
WPPSI-III: Wechsler Preschool and Primary Scale of Intelligence test 3rd UK edition
