# Supplementary material for: Effect of continued folic acid supplementation beyond the first trimester of pregnancy on cognitive performance in the child: a follow-up study from a randomized controlled trial (FASSTT Offspring Trial)
Source: BMC Med. 2019 Oct 31;17:196. doi: 10.1186/s12916-019-1432-4 (PMC6823954; doi:10.1186/s12916-019-1432-4)
Supplement: Supplementary file 1 — Additional file 1: Table S1a. Characteristics of responders and non-responders to participation in the FASSTT Offspring trial at 7 years. Table S1b. Characteristics of non-responders to participation in the FASSTT Offspring trial at 7 years by treatment group. Table S2. Anthropometric measurements of FASSTT Offspring trial participants at age 3 and 7 years. Table S3. WPPSI -III test scores of FASSTT Offspring trial participants at 7 years. Table S4a. Maternal serum folate status at 36th GW and WPPSI-III test scores of FASSTT Offspring trial participants at 7 years. Table S4b. Maternal RBC folate status at 36th GW and WPPSI-III test scores of FASSTT Offspring trial participants at 7 years. [file 12916_2019_1432_MOESM1_ESM.docx]

**Table S1a.** Characteristics of responders and non-responders to participation in the FASSTT Offspring trial at 7 years

| Maternal Characteristics | Responders (*n* = 70) | Non-responders (*n* = 49) | *p* value |
| --- | --- | --- | --- |
| Age, y^a^ | 28.7 (27.8, 29.7) | 27.8 (26.5, 29.0) | 0.207 |
| BMI, kg/m^2^ | 24.9 (23.7, 26.0) | 24.3 (23.3, 25.4) | 0.512 |
| Smoking in pregnancy, % | 16 | 27 | 0.127 |
| Alcohol use, % | 3 | 7 | 0.262 |
| Iron supplement use, % | 23 | 23 | 0.962 |
| Week of gestation at labor | 40.0 (39.7, 40.4) | 39.9 (39.6, 40.2) | 0.587 |
| Parity, *n* | 0.8 (0.6, 1.1) | 1.1 (0.8, 1.3) | 0.198 |
| Child Characteristics at birth |  |  |  |
| Sex, female % | 54 | 52 | 0.921 |
| Birth weight, g | 3442 (3310, 3575) | 3485 (3368, 3601) | 0.642 |
| Birth length, cm | 51.1 (50.5, 51.7) | 50.5 (49.9, 51.2) | 0.202 |
| Head circumference, cm | 34.6 (34.2, 35.0) | 34.6 (34.2, 34.9) | 0.858 |
| Apgar score at 1^st^ minute | 8.6 (8.4, 8.8) | 8.5 (8.2, 8.8) | 0.621 |
| Apgar score at 5^th^ minute | 9.0 (8.9, 9.1) | 9.0 (8.8, 9.1) | 0.760 |
| Breastfed from birth, % | 43 | 36 | 0.415 |

Values presented as mean (95% CI). Data analyzed by independent t-test (continuous variables) or chi-square (categorical variables). P < 0.05 considered significant.

^a^Refers to age of mother at enrolment to the FASSTT trial.

**Table S1b.** Characteristics of non-responders to participation in the FASSTT Offspring trial at 7 years by treatment group

| Maternal Characteristics | Placebo (*n =* 27) | Folic Acid (*n* = 22) | *p* value |
| --- | --- | --- | --- |
| Age, y^a^ | 27.0 (25.4, 28.6) | 28.9 (26.9, 30.9) | 0.131 |
| BMI, kg/m^2^ | 24.2 (22.8, 25.6) | 24.5 (22.6, 26.4) | 0.808 |
| Smoking in pregnancy, % | 32 | 18 | 0.242 |
| Alcohol use, % | 9 | 5 | 0.544 |
| Iron supplement use, % | 24 | 23 | 0.945 |
| Week of gestation at labor | 40.1 (39.8, 40.4) | 39.6 (39.1, 40.1) | 0.080 |
| Parity, *n* | 1.1 (0.7, 1.5) | 1.1 (0.7, 1.4) | 0.963 |
| Child Characteristics at birth |  |  |  |
| Sex, female % | 62 | 36 | 0.063 |
| Birth weight, g | 3436 (3291, 3582) | 3560 (3354, 3765) | 0.304 |
| Birth length, cm | 50.5 (49.7, 51.4) | 50.5 (49.6, 51.5) | 0.980 |
| Head circumference, cm | 34.4 (34.0, 34.9) | 34.8 (34.3, 35.3) | 0.259 |
| Apgar at 1^st^ minute | 8.5 (8.2, 8.9) | 8.4 (7.7, 9.1) | 0.716 |
| Apgar at 5^th^ minute | 9.0 (8.9, 9.1) | 8.9 (8.6, 9.2) | 0.481 |
| Breastfed from birth, % | 35 | 36 | 0.935 |

Values presented as mean (95% CI). Data analyzed by independent t-test (continuous variables) or chi-square (categorical variables). *P* < 0.05 considered significant.

^a^Refers to age of mother at enrolment to the FASSTT trial.

**Table S2.** Anthropometric measurements of FASSTT Offspring trial participants at age 3 and 7 years

| Anthropometric Measurements | Children at 3 years (*n* = 39) | |  | Children at 7 years (*n* = 70) | |
| --- | --- | --- | --- | --- | --- |
|  | Placebo (*n* = 16) | Folic Acid (*n* = 23) |  | Placebo (*n*= 33) | Folic Acid (*n*= 37) |
| Weight, kg | 15.2 (13.1-17.3) | 14.2 (13.5-15.0) |  | 24.1 (22.6-25.7) | 22.8 (21.7-24.0) |
| Height, cm | 93.6 (90.7-96.5) | 94.2 (92.5-95.9) |  | 118.5 (112.0-124.9) | 120.4 (118.3-122.5) |
| BMI Z score^a^ | 0.55 (-0.39-1.48) | 0.19 (-0.19-0.57) |  | 0.24 (-0.11-0.58) | -0.08 (-0.40-0.23) |
| Waist circumference, cm | 51.6 (46.9-56.4) | 50.3 (49.0-51.7) |  | 55.6 (54.1-57.1) | 54.3 (53.0-55.6) |
| Head circumference, cm | NR^b^ | NR |  | 52.6 (51.5-53.7) | 51.7 (50.9-52.6) |
| Body fat, %^c^ | NR | NR |  | 15.8 (13.5-18.1) | 15.3 (13.6-17.0) |

Data are expressed as mean (95% CI).

^a^BMI for age Z scores were calculated using the World Health Organization Anthro Software package (version 3.2.2; Geneva, WHO).

^b^Not recorded.

^c^For children aged 7 years, body fat measures were also obtained using the Tanita-305 body fat analyzer (Tanita Corp, Tokyo, Japan).

**Table S3:** WPPSI-III test scores of FASSTT Offspring trial participants at 7 years^a^

| Composite  and subtest scores | Placebo (n=33) | Folic acid (n=37) | Difference | P value (Unadjusted)^b^ | P value (Adjusted)^c^ |
| --- | --- | --- | --- | --- | --- |
| Verbal IQ | 103.4 (99.4, 107.4) | 107.7 (103.7, 111.8) | 4.3 (-1.2, 9.9) | 0.126 | 0.120 |
| Information | 10.9 (9.9, 11.8) | 11.1 (10.3, 12.0) | 0.3 (-1.0, 1.5) | 0.648 | 0.630 |
| Vocabulary | 9.6 (8.9, 10.4) | 10.3 (9.5, 11.1) | 0.7 (-0.4, 1.8) | 0.221 | 0.262 |
| Word Reasoning | 11.9 (11.0, 12.8) | 13.3 (12.4, 14.2) | 1.4 (0.2, 2.6) | 0.023 | 0.027 |
| Performance IQ | 100.6 (96.5, 104.6) | 104.1 (99.1, 109.1) | 3.5 (-2.9, 9.9) | 0.274 | 0.429 |
| Block Design | 9.6 (8.8, 10.4) | 10.2 (9.4, 11.1) | 0.6 (-0.5, 1.8) | 0.282 | 0.354 |
| Matrix Reasoning | 10.2 (9.5, 11.0) | 11.1 (10.2, 12.1) | 0.9 (-0.3, 2.1) | 0.151 | 0.408 |
| Picture Concepts | 10.8 (9.7, 11.8) | 11.0 (9.8, 12.1) | 0.2 (-1.4, 1.7) | 0.838 | 0.900 |
| Processing Speed | 103.9 (98.1, 109.7) | 102.5 (97.4, 107.7) | 1.4 (-6.2, 9.0) | 0.718 | 0.712 |
| Symbol Search | 10.5 (9.7, 11.4) | 10.7 (9.8, 11.6) | 0.2 (-1.1, 1.4) | 0.773 | 0.750 |
| Coding | 11.1 (9.6, 12.6) | 10.4 (9.1, 11.7) | 0.7 (-1.2, 2.6) | 0.466 | 0.423 |
| General Language | 105.8 (101.1, 110.5) | 108.9 (104.5, 113.2) | 3.1 (-3.2, 9.4) | 0.334 | 0.514 |
| Receptive Vocab | 11.2 (10.2, 12.1) | 11.7 (10.9, 12.5) | 0.5 (-0.7, 1.7) | 0.384 | 0.497 |
| Picture Naming | 11.1 (10.1, 12.1) | 11.7 (10.7, 12.7) | 0.6 (-0.9, 2.0) | 0.434 | 0.625 |
| Full Scale IQ | 103.5 (99.3, 107.6) | 106.4 (101.7, 111.1) | 3.0 (-3.3, 9.2) | 0.348 | 0.441 |
| *Additional Subtests* |  |  |  |  |  |
| Comprehension | 10.2 (9.4, 11.1) | 10.0 (9.3, 10.7) | 0.2 (-0.8, 1.3) | 0.647 | 0.405 |
| Picture Completion | 10.2 (8.9, 11.5) | 11.4 (10.3, 12.6) | 1.2 (-0.5, 2.9) | 0.155 | 0.209 |
| Similarities | 10.2 (9.6, 10.7) | 10.9 (10.1, 11.7) | 0.7 (-0.2, 1.7) | 0.129 | 0.129 |
| Object Assembly | 11.3 (10.3, 12.4) | 12.0 (11.4, 12.7) | 0.7 (-0.5, 1.9) | 0.267 | 0.303 |

Data presented as mean (95% CI).

^a^Test scores assessed by Wechsler Preschool and Primary Scale of Intelligence test, 3^rd^ UK edition (WPPSI-III).

Data analyzed by ^b^independent *t*, test and ^c^ANCOVA, with adjustment for child’s sex, birth weight, breastfeeding, maternal age and maternal education attainment. Results considered significant when *P* < 0.05.

**Table S4a.** Maternal serum folate status at 36^th^ GW and WPPSI-III test scores of FASSTT Offspring trial participants at 7 years

|  | Verbal IQ^1^ | | | | Performance IQ | | | | | Processing Speed | | | | General Language | | | | Full Scale IQ | | | |
| --- | --- | --- | --- | --- | --- | --- | --- | --- | --- | --- | --- | --- | --- | --- | --- | --- | --- | --- | --- | --- | --- |
|  | β | B | 95% CI | *P*  value | β | B | 95% CI | *P*  value | β | | B | 95% CI | *P*  value | β | B | 95% CI | *P*  value | β | B | 95% CI | *P*  value |
| Serum folate at 36 GW^2^ | 0.268 | 0.001 | 0.000, 0.001 | 0.027 | -0.116 | -0.064 | -0.195, 0.067 | 0.333 | -0.059 | | -0.038 | -0.208, 0.131 | 0.653 | 0.106 | 0.058 | -0.069, 0.184 | 0.366 | -0.007 | -0.004 | -0.135, 0.128 | 0.957 |
| Maternal age | -0.022 | -2.6E-4 | -0.003, 0.003 | 0.857 | 0.059 | 0.731 | -0.067, 1.529 | 0.641 | -0.027 | | -0.102 | -1.135, 0.930 | 0.843 | 0.058 | 0.183 | -0.587, 0.954 | 0.636 | 0.129 | 0.405 | -0.393, 1.204 | 0.314 |
| Breastfed | 0.300 | 0.029 | 0.005, 0.053 | 0.017 | 0.220 | 5.956 | -0.643, 12.555 | 0.086 | 0.164 | | 5.163 | -3.381, 13.706 | 0.231 | 0.369 | 9.769 | 3.397, 16.142 | 0.003 | 0.314 | 8.230 | 1.622, 14.837 | 0.016 |
| Maternal education attainment | 0.143 | 0.003 | -0.002, 0.007 | 0.245 | 0.177 | 0.754 | -0.440, 1.948 | 0.157 | -0.135 | | -0.771 | -2.317, 0.775 | 0.323 | 0.008 | 0.038 | -1.115, 1.191 | 0.948 | 0.080 | 0.380 | -0.816, 1.576 | 0.528 |
| Child’s sex (M) | 0.003 | 2.6E-4 | -0.023, 0.024 | 0.982 | 0.168 | -7.377 | -13.843, -0.911 | 0.173 | -0.083 | | -2.604 | -10.975, 5.768 | 0.536 | -0.248 | -6.527 | -12.771, -0.283 | 0.041 | -0.153 | -3.989 | -10.464, 2.485 | 0.223 |

^1^Multiple linear regression analysis performed with maternal serum folate status at 36^th^ GW and WPPSI-III test scores as the dependent variables. The analysis has been adjusted for relevant covariates (maternal age, breastfeeding, maternal education attainment and child’s sex). β, standardized coefficient; B, unstandardized coefficient; GW, gestational week. *P <* 0.05 was considered significant.

^2^36th GW refers to postintervention.

**Table S4b.** Maternal RBC folate status at 36^th^ GW and WPPSI-III test scores of FASSTT Offspring trial participants at 7 years

|  | Verbal IQ^1^ | | | | Performance IQ | | | | Processing Speed | | | | General Language | | | | Full Scale IQ | | | |
| --- | --- | --- | --- | --- | --- | --- | --- | --- | --- | --- | --- | --- | --- | --- | --- | --- | --- | --- | --- | --- |
|  | β | B | 95% CI | *P*  value | β | B | 95% CI | *P*  value | β | B | 95% CI | *P*  value | β | B | 95% CI | *P*  value | β | B | 95% CI | *P*  value |
| RBC folate at 36 GW^2^ | 0.250 | 1.7E-5 | 2.2E-7, 3.4E-5 | 0.047 | 0.102 | 0.002 | -0.003, 0.007 | 0.426 | -0.100 | -0.002 | -0.008, 0.004 | 0.460 | 0.103 | 0.002 | -0.003, 0.007 | 0.414 | 0.082 | 0.002 | -0.003, 0.006 | 0.517 |
| Maternal age | -0.067 | -0.001 | -0.004, 0.002 | 0.591 | 0.059 | 0.191 | -0.624, 1.007 | 0.641 | -0.063 | -0.239 | -1.257, 0.779 | 0.641 | -0.047 | -0.150 | -0.943, 0.643 | 0.706 | 0.018 | 0.057 | -0.730, 0.845 | 0.885 |
| Breastfed | 0.293 | 0.029 | 0.005, 0.053 | 0.020 | 0.220 | 5.927 | -.0870, 12.724 | 0.086 | 0.194 | 6.120 | -2.365, 14.605 | 0.154 | 0.398 | 10.532 | 3.921, 17.143 | 0.002 | 0.323 | 8.456 | 1.892, 15.019 | 0.012 |
| Maternal education attainment | 0.114 | 0.002 | -0.002, 0.006 | 0.346 | 0.177 | 0.865 | -0.344, 2.074 | 0.157 | -0.133 | -0.758 | -2.267, 0.751 | 0.319 | 0.004 | 0.019 | -1.157, 1.195 | 0.974 | 0.083 | 0.394 | -0.773, 1.561 | 0.502 |
| Birth weight | 0.134 | 1.2E-5 | -9.1E-6, 3.3E-5 | 0.262 | 0.168 | 0.004 | -0.002, 0.010 | 0.173 | 0.110 | 0.003 | -0.004, 0.010 | 0.403 | 0.101 | 0.002 | -0.003, 0.008 | 0.405 | 0.189 | 0.004 | -0.001, 0.010 | 0.124 |

^1^Multiple linear regression analysis performed with maternal RBC folate status at 36^th^ GW and WPPSI-III test scores as the dependent variables. The analysis has been adjusted for relevant covariates (maternal age, breastfeeding, maternal education attainment and birth weight). β, standardized coefficient; B, unstandardized coefficient; GW, gestational week; RBC, red blood cell. *P <* 0.05 was considered significant.

^2^36th GW refers to postintervention.
